# Gabapentin prevalence: clinical and forensic experience in St. Louis, Missouri, USA

**DOI:** 10.1080/20961790.2021.1991075

**Published:** 2021-11-19

**Authors:** Sarah B. Riley, Kelsie Garbutt, Chelsea Crow, T. Scott Isbell, Anthony J. Scalzo

**Affiliations:** a School of Medicine Department of Pathology, Saint Louis University, St. Louis, Missouri, USA; b School of Medicine Department of Pediatrics, Saint Louis University, St. Louis, Missouri, USA; c Department of Sociology and Anthropology, Forensic Sciences Program, Saint Louis University, St. Louis, Missouri, USA

**Keywords:** Forensic toxicology, clinical toxicology, substance misuse, gabapentin, urine drug screening, postmortem, mass spectrometry

## Abstract

Gabapentin (Neurontin) is an anti-epileptic drug that has had wide off-label prescription use since market release due to presumed negligible abuse potential. However, trends in drug misuse have demonstrated that gabapentin misuse is occurring, particularly in those with a history of opioid misuse. This is concerning, because although gabapentin has no direct ligand activity at opioid receptors, it does potentiate the analgesic effect of opioids, and concurrent use of gabapentin and opioids may increase the risk of respiratory depressive effects of opioids. This study investigates the incidence of gabapentin detected in urine samples collected for clinical drug screening purposes in a local hospital emergency department and in postmortem samples submitted by medical examiners in the St. Louis metropolitan area. The prevalence of gabapentin and co-detected drugs in both populations is contrasted, compared, and discussed. This study found that 30% of urine samples collected from patients with suspected drug intoxication presenting to SSM Health Saint Louis University Hospital, a quaternary care medical center, were positive for gabapentin, and nearly two thirds of those were also positive for oxycodone. Over a 6-month period, the incidence of gabapentin positive postmortem cases increased from 18% to 20%. Nearly all gabapentin positive postmortem cases were also positive for an opioid, the most significant being fentanyl, suggesting that gabapentin misuse may be due to its potentiating effect of opioid drug action. This study also highlights the limited utility of immunoassay-based urine drug screens.

## Introduction

Gabapentin (Neurontin) is a GABA analog that does not bind to GABA(A), GABA(B), benzodiazepine, or cannabinoid receptors. However, some evidence exists in a murine model that gabapentin may enhance expression of the γ-subunit of the GABA(A) complex and lead to tonic inhibition of neuronal conductance [1]. Use of gabapentin results in increased concentrations of GABA and possibly decreased concentrations of glutamate, although in a clinical study no effect on glutamate levels was seen [2]. Gabapentin was first approved by the US Food and Drug Administration (FDA) in 1993 for treatment of epilepsy, and in 2004 was approved for the treatment of post-herpetic neurologic pain. The mechanism of gabapentin-mediated analgesia remains unclear, although a subunit of the voltage gated calcium channel complex has been identified as a gabapentin binding protein [3]. Since its approval for neuropathic pain, gabapentin has had wide off-label prescription use with some estimating nearly 95% of gabapentin prescription being off-label [4]. Some off-label uses include various neuropathic pain conditions, mental health disorders, migraines, drug and alcohol addiction, and general pain [5, 6].

Reasons behind the wide off-label prescription use include the drug’s safety and presumed negligible abuse potential. However, trends in drug misuse have demonstrated that gabapentin misuse is occurring, particularly in those with a history of opioid misuse. This is concerning, because although gabapentin has no activity at opioid receptors, it does potentiate the analgesic effect of opioids and concurrent use of gabapentin and opioids can increase the risk of respiratory depressive effects of opioids [7]. In fact, in 2014 a product monograph was amended to warn against the possibility of respiratory depression when combining gabapentin with opioids [8].

Gabapentin is not frequently included in standard drug screening tests used in clinical (hospital) settings in the US. Urine drug screening of 7–9 drug classes by immunoassay is commonly used in hospital laboratories for rapid screening of patients with suspected drug use. Immunoassays are relatively inexpensive and easy to use, requiring little to no sample preparation and easy to interpret data. However, immunoassays are not easy to adapt to rapidly changing drug climates. This study compared results from standard urine drug screening at a city hospital to mass spectrometry-based testing at a forensic toxicology laboratory. Trends discovered in the clinical samples were compared to trends in the postmortem drug testing routinely performed in the forensic laboratory. Gabapentin was a significant finding in both clinical and forensic cases. Gabapentin was not included in the urine drug screen panel at used in the hospital laboratory. Gabapentin was co-detected with opioids in most cases.

## Materials and methods

Urine drug screening at the SSM Health Saint Louis University Hospital Laboratory was performed on an Architect ci 8200 (Abbott, Chicago, IL, USA) immunoassay platform. The following eight drug classes were included in the screen: amphetamine/methamphetamine, barbiturates, methadone, phencyclidine, opiates, cannabinoids, cocaine, and benzodiazepines. The assays were homogenous enzyme immunoassays using proprietary, ready-to-use reagents. Briefly, drug assays used glucose-6-phosphate dehydrogenase or β-galactosidase and the activity of either enzyme, as measured by absorption of either NADH (glucose-6-phosphate) at 340/416 nm or chlorophenol red (β-galactosidase) at 570 nm, as a qualitative indicator of antigen (drug) present. Patient urine aliquots were loaded directly on to the instrument with no further manipulation. Detection limits for each drug class are listed in Table 1.

**Table 1.** Analytical limit of detection (LOD) of drug classes detected by immunoassay (UDS) compared to mass spectrometry (MS). While individual drugs in a class (e.g. morphine, codeine, hydrocodone) are detected by MS *vs*. a simple positive for class (e.g. opiates), the LOD for the MS method listed in the table represents the highest LOD for the appropriate drug group detected by MS.

Drug class	LOD – UDS (ng/mL)	LOD – MS (ng/mL)
Amphetamines	1000	10
Barbiturates	200	100
Benzodiazepines	200	20
Opiates*	300	5
Phencyclidine	25	5
Cannabinoids	50	5
Methadone	300	20
Cocaine	300	50

* This group does not include the semi-synthetic opioid, oxycodone.

For analysis performed at the Saint Louis University Forensic Toxicology Laboratory, all reagents were purchased from Sigma Aldrich (St. Louis, MO, USA). All drug standards were purchased from Cerillant Analytical Reference Standards (Round Rock, TX, USA). One hundred urine specimens previously collected by emergency medicine physician order and analyzed by immunoassay-based drug screen at SSM Health Saint Louis University Hospital were de-identified and submitted to the Saint Louis University Forensic Toxicology for analysis by a targeted liquid chromatography tandem mass spectrometry method. The mass spectrometry panel included 89 drug and drug metabolites with limits of detection ranging from 0.1 ng/mL to 200 ng/mL (Supplementary Table S1). Analysis was performed on an ABSciex 4500 Triple Quadrupole Mass Spectrometer (MS) with an ABSciex Exion Liquid Chromatography (LC) system. A 2.6 μm biphenyl 100 A, 50 × 4.6 mm reverse phase chromatography column (Phenomenex, Torrance, CA, USA) was used for compound separation. Internal standards were used for each analyte and method recovery was greater than 70% for all analytes included in the method panel. Retention times and two multiple reaction monitoring transitions (*m*/*z* → *m*/*z*) for each compound were used for identification. The MS method involved polarity switching to detect both positive and negative ions in the same method. An LC gradient was developed, resulting in baseline resolution of all compounds in a 7-min total run time. The method was validated per Scientific Working Group for Forensic Toxicology (SWG-TOX) guidelines (2018). Two hundred and fifty μL of each urine sample was prepared for analysis by protein precipitation with 750 μL of acetone. After brief centrifugation, the samples were evaporated to dryness with nitrogen gas, and reconstituted in 100 μL of methanol. Reconstituted specimens were loaded on to the LC-MS system for analysis.

This same method and panel of compounds was used for analysis of routine postmortem casework submitted to the Saint Louis University Forensic Toxicology Laboratory.

## Results

**Figure 1.**
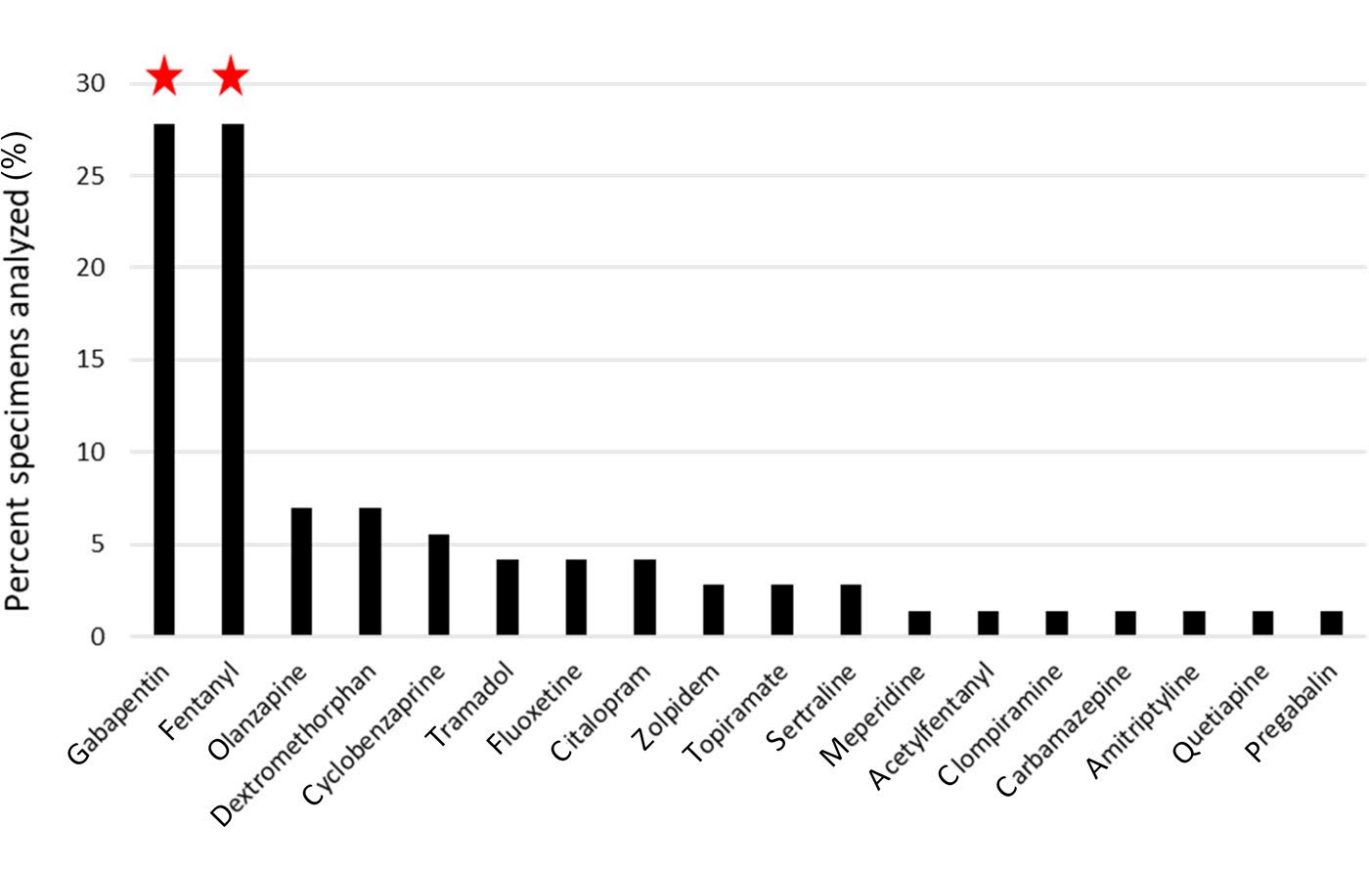
Drugs not included in the hospital immunoassay-based drug screen that were detected by LC-MS/MS. Several significant drugs were detected by mass spectrometry in urine samples collected in the Saint Louis University Hospital Emergency Department, that were not detected by the hospital’s immunoassay-based urine drug screen. Including: fentanyl, gabapentin, olanzapine, dextromethorphan, cyclobenzaprine, tramadol, fluoxetine, citalopram, zolpidem, sertraline, meperidine, acetyl-fentanyl, clomipramine, carbamazepine, amitriptyline, quetiapine, and pregabalin. Fentanyl and gabapentin were the most frequently detected. For brevity, only parent compounds detected are reported here. Metabolites of many of these drugs were also detected, but not included in this graph.

Analysis of the urine specimens collected from the hospital by tandem mass spectrometry showed many significant drugs, previously not detected by the immunoassay-based urine drug screen, including: fentanyl, gabapentin, olanzapine, cyclobenzaprine, tramadol, fluoxetine, citalopram, zolpidem, topiramate, sertraline, meperidine, acetylfentanyl, clomipramine, carbamazepine, amitriptyline, quetiapine, and pregabalin (Figure 1). Fentanyl and gabapentin were the most significant findings; these drugs were detected in 28% of the specimens. Of the 28% of urine samples in which gabapentin was detected, 95% were also positive for one or more opioid. Gabapentin was co-detected with fentanyl in 33% of the specimens, with oxycodone in 33% of the samples, and with fentanyl plus one or more non-fentanyl opioid in 33% of the samples. Of the non-fentanyl opioids detected, oxycodone was the most common opioid co-detected with gabapentin with an incidence of 50%. Morphine, 6-monoacetylmorphine, and/or codeine, compounds consistent with heroin use, were detected in 27% of gabapentin positive cases but always with fentanyl or oxycodone co-detected. Oxycodone was not included in the immunoassay-based urine drug screen either.

**Figure 2.**
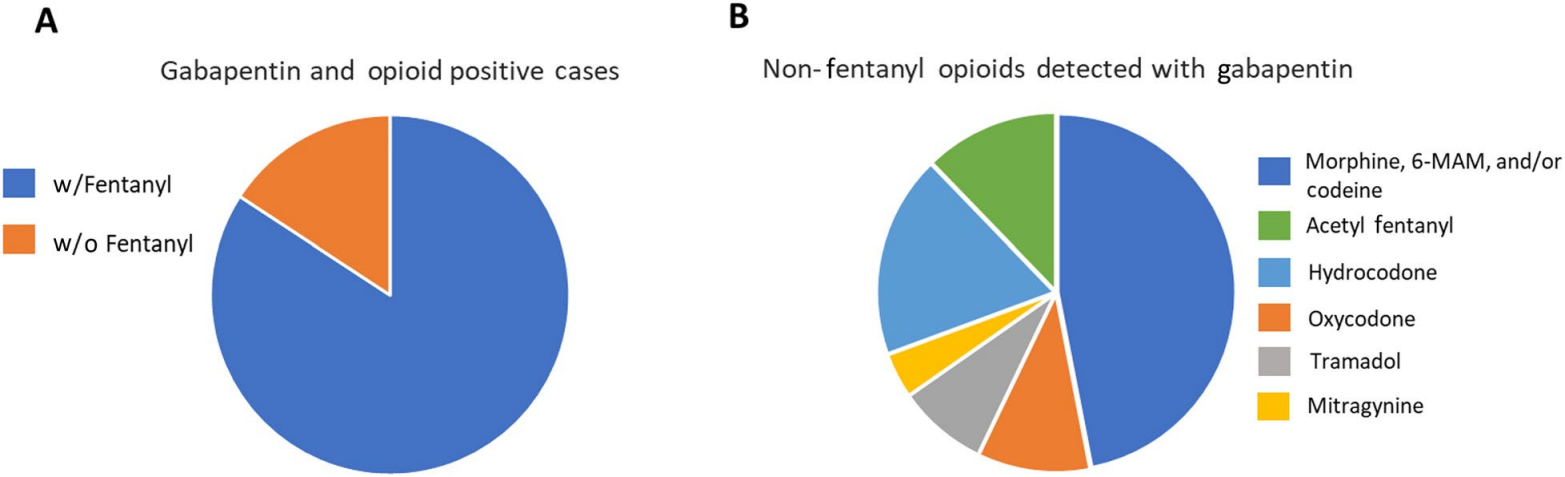
Distribution of fentanyl and non-fentanyl opioids co-detected with gabapentin in postmortem casework submitted to the Saint Louis University Forensic Laboratory, October–December 2020. (A) Distribution of gabapentin positive cases where fentanyl was co-detected with gabapentin (w/fentanyl) and cases where gabapentin was detected with a non-fentanyl opioid (w/o fentanyl). Fentanyl was detected in most cases wherein gabapentin and an opioid were detected. (B) Distribution of non-fentanyl opioids co-detected with gabapentin. Morphine, 6-MAM, and codeine were the most frequent non-fentanyl opioid detected with gabapentin.

Retrospective analysis of reported results in postmortem cases from October–December 2019 was performed (Figure 2). Routine toxicological evaluation of 299 postmortem cases with positive toxicology results were reviewed to the determine the incidence of gabapentin. The cause of death (i.e. opioid overdose) was not considered in the review. Gabapentin was detected in 27% of the positive casework. In 91% of cases wherein gabapentin was detected, an opioid was also detected. Fentanyl and one or more opioids, including acetyl fentanyl, were co-detected with gabapentin in 55% of the postmortem cases. Gabapentin was co-detected with a non-fentanyl opioid in only 9% of cases. Oxycodone was only found without fentanyl in 3% of case. Cocaine was the most common non-opioid drug detected with gabapentin in postmortem casework; it was found in 31% of cases in which gabapentin was also detected. Gabapentin was not detected with cocaine in clinical urine samples. Morphine, 6-monoacetylmorphine, and codeine were detected in postmortem casework at a rate of 23%. In 6% postmortem cases in which gabapentin was detected but no other drug was co-detected, ethanol was determined to be present. Ingestion of ethanol (*versus in-vitro* synthesis or contamination) was proved by the presence of ethanol in vitreous fluid associated with these cases.

## Discussion

Prescription drug trends in the US, Canada, and Europe indicate an increase in gabapentin use for a wide array of indications including on-label use as an adjuvant in epilepsy therapy and post-herpetic neuropathic pain, as well as broad off-label use for general chronic pain, neuropathic pain, migraines, and mental health disorders [4, 9]. A study of incidence of gabapentin prescriptions in a commercially insured adult population in the US showed that the rate of gabapentin prescriptions doubled from 2009–2016 [10].

Perception of low abuse potential as well as efforts to avoid prescribing opioids have likely been a contributing factor in these trends [11]. However, there is increasing evidence of misuse and diversion, particularly among individuals misusing opioids. A 2017 study showed that while the incidence of gabapentin misuse in the general population of 1%, the incidence among those misusing opioids is as high as 68% [12]. The rate at which gabapentin in misused in conjunction with opioids is alarming due to the opioid-potentiating effect of gabapentin and the increased risk of severe respiratory depression when gabapentin is used in conjunction with opioids [13]. In 2015, Kentucky reported that 41% of fatal polysubstance overdoses were positive for gabapentin [10]. A 2018 investigation of the prevalence of gabapentin detected in drug overdose cases in certain jurisdictions of Tennessee, West Virginia, North Carolina, and Kentucky found that, on average, gabapentin was co-detected in 26% of cases where an opioid was also detected. The study also showed geographical variation in the rate of gabapentin and opioid co-detection, varying from 4% in Northeast Tennessee to 41% in Kentucky [14].

In the study presented here, we evaluated the incidence of significant drugs in urine collected in the emergency department of an urban hospital, that were not detected by the immunoassay-based urine drug screen performed in the hospital laboratory, by re-analyzing specimens on a more specific and sensitive MS based method used at the Saint Louis University Forensic Toxicology Laboratory. Significant drugs were defined as those that have the potential to cause toxicity and physician knowledge of the result would impact patient management. This study began as an internal quality improvement project with the goal of determining how best to increase the scope of toxicology screening in the hospital laboratory.

Immunoassay methods are widely used in clinical setting due to relatively low cost and ease of use and result interpretation, but there are drawbacks to relying solely on these methods. Firstly, while monoclonal antibodies are used in these assays, the antibodies are developed to target the largest or most common drug or drug metabolite in a drug class. For example, the antibody used in the benzodiazepine immunoassay is targeted to oxazepam, and as such has very poor cross-reactivity with benzodiazepines that do not produce this metabolite, including alprazolam and clonazepam. Secondly, the cut-offs of immunoassays for urine drug screening are targeted to SAMSHA guidelines for DOT drug testing; designed to minimized false positives and without consideration to clinical or public health applications. Thirdly, due to the very nature of the assay, immunoassays are not dynamic and thus difficult to modify as new drug misuse trends emerge. In contrast, drug screening by mass spectrometry is both sensitive and specific, identifying the exact drug or drug metabolite present in a sample by chemical properties (retention time on a chromatography column), molecular weight, and ion fragmentation. Also, mass spectrometry methods can be rapidly edited to include or eliminate compounds to reflect changes in drug misuse trends. However, in comparison to immunoassay platforms, mass spectrometry systems are costly and require technical expertise.

Not surprisingly, several significant drugs were detected in the urine specimens by targeted tandem mass spectrometry-based assay, that were not detected by the immunoassay platform (Figure 1). Reasons for the discrepancy include 1) difference in analytical cut-off between the two methods, and 2) the lack of inclusion of the drug (or drug class) in the immunoassay panel.

Significant drugs determined by the MS method included anticholinergics (e.g. amitriptyline, clomipramine), antiepileptics and antipsychotics (e.g. fluoxetine, carbamazepine, quetiapine, gabapentin), and central nervous system depressants (e.g. fentanyl, dextromethorphan, tramadol, merperidine, zolpidem). All of these drugs have been associated with toxicity, and a positive drug screen combined with the patient presentation may aid physicians in the medical management of an intoxicated patient. However, the most outstanding findings of our study was the noticeable prevalence of fentanyl and gabapentin detected during the re-analysis.

Due to the potential for gabapentin use in conjunction with opioids, the data were further reviewed to determine the incidence of opioids co-detected with gabapentin, and to identify the opioids (if any) detected with gabapentin. Remarkably, opioids were detected in nearly 100% of the urine specimens wherein gabapentin was detected. The opioids co-detected with gabapentin were nearly an even distribution of fentanyl, a non-fentanyl opioid, or fentanyl and a non-fentanyl opioid. Oxycodone was the most frequent non-fentanyl opioid detected with gabapentin. These findings are consistent with reports from other states, and trends of higher incidence of gabapentin use among individuals using an opioid (either prescription or non-prescription use). From a patient management standpoint, these findings are very significant. A drug seeking individual presenting to the emergency department complaining of pain could screen negative for drugs and be prescribed gabapentin, with the physician aiming to avoid opioid prescribing and naïve to misuse of gabapentin, fentanyl, or oxycodone. Illicit fentanyl and oxycodone are both available in the St. Louis area.

Based on the increased risk of fatal respiratory event when gabapentin is used with opioids, prevalence of gabapentin and its co-detection with one or more opioids was evaluated postmortem casework (Figure 2). As of December 2020, gabapentin is the 9th most frequently detected drug in casework submitted to the laboratory, with 21.7% of all cases positive for gabapentin. This is very similar to the incidence in the urine samples collected from the emergency department, as well as to the average incidence among Tennessee, North Carolina, West Virginia, and Kentucky reported in 2018 by Slovava et al. [14]. Also, like the findings in the clinical urine specimens, cases positive for gabapentin were most frequently detected with one or more opioids, with fentanyl being the most commonly detected. The rate of detection of morphine, 6-monoacetylmorphine, and/or codeine was also similar between the two study populations. These three compounds detected together are highly consistent with heroin use, but when 6-monoacetyl- morphine is not detected, heroin as the sole source of morphine and codeine cannot be presumed. Unlike findings in the clinical urine samples, oxycodone was not detected as the only opioid in many of the postmortem cases, in fact, oxycodone was the only opioid co-detected with gabapentin in 3% of the postmortem cases compared to the 30% positive rate in the clinical sample population. Compared to the clinical samples, there was more variation in the opioids detected in the postmortem samples, to include tramadol, hydrocodone, and even mitragynine. It may be that individuals succumbing to polydrug overdoses involving gabapentin are more likely to use mostly street drugs (illicit fentanyl and heroin) then to use an opioid like oxycodone whether that is obtained by personal prescription or illicit means.

Also, in contrast to the clinical urine specimens, analysis of the postmortem cases demonstrated an increased incidence of cocaine in gabapentin-positive cases. This is another indicator of gabapentin use in the substance misuse population. Finally, a small number of cases had only gabapentin and ethanol detected.

This study has some limitations. Initially designed as a quality improvement project, this study used only de-identified samples and did not investigate whether the drugs detected were prescribed to the patient. Also, due to the nature of the study, we were not able to correlate the number of patients presenting with suspected drug intoxication/overdose or the number of cases with overdose as the assigned cause of death to the incidence of gabapentin detection. Further work should continue to determine the prevalence of gabapentin involvement in drug overdose deaths in the St. Louis region.

In summary, this study provides preliminary evidence of dangerous gabapentin misuse in the St. Louis, Missouri metropolitan region. Nearly 30% of patients presenting to the SSM Health Saint Louis University Hospital Emergency Department were, based on urine toxicology testing, positive for gabapentin and one or more opioids. This incidence is essentially identical to the number of postmortem cases submitted to the Saint Louis University Forensic Toxicology Laboratory. In both populations, fentanyl was the most frequently encountered opioid, followed by morphine, 6-monoacetyl fentanyl, and/or codeine, and oxycodone. At the time of the study, neither fentanyl nor gabapentin was included in the immunoassay-based urine drug screen used by the hospital. It is possible that individuals presenting to the hospital may have been drug seeking and discharged with prescriptions for gabapentin based on a “clean” drug screen. These individuals might not survive subsequent polydrug overdoses including gabapentin and opioids. Without knowledge of gabapentin misuse in patients presenting to the emergency department, physicians lack the opportunity (1) to avoid gabapentin prescription to patients misusing the drug, and (2) to counsel patients on the dangers of concurrent use of gabapentin and opioids. This study also highlights the limitations of immunoassay-based urine drug screens for identifying and managing drug misuse trends. During the preparation of this manuscript, fentanyl has been added to the urine drug screen at Saint Louis University Hospital, but gabapentin is still not included.

## Supplementary Material

Supplemental Material
