# Supplementary material for: Gabapentin prevalence: clinical and forensic experience in St. Louis, Missouri, USA
Source: Forensic Sci Res. 2021 Nov 19;6(3):218–23. doi: 10.1080/20961790.2021.1991075 (PMC8635626; doi:10.1080/20961790.2021.1991075)
Supplement: Supplemental Material [file TFSR_A_1991075_SM7272.docx]

Supplementary Table 2. Drugs and drug metabolites detected by the mass spectrometry (MS) method, with corresponding cut-offs.

| Compound | Reporting Limit (ng/mL) | Compound | Reporting Limit (ng/mL) |
| --- | --- | --- | --- |
| 2-furanyl fentanyl | 0.1 | Levetiracetam | 200 |
| 6-monoacetylmorphine | 5 | Lorazepam | 5 |
| 7-aminoclonazepam | 5 | MDA | 10 |
| Acetaminophen | 100 | MDEA | 10 |
| Acetyl fentanyl | 0.1 | MDMA | 10 |
| Alprazolam | 5 | MDPV | 10 |
| AM-2201 4-OH-pentyl | 5 | Meperidine | 10 |
| Amitriptyline | 20 | Meprobamate | 20 |
| Amo/pentobarbital^*^ | 100 | Methadone | 20 |
| Amphetamine | 10 | Methamphetamine | 10 |
| Benzoylecgonine | 50 | Methylphenidate | 10 |
| Buprenorphine | 0.5 | Midazolam | 5 |
| Bupropion | 10 | Mitragynine (Kratom) | 10 |
| Butabarbital | 100 | Morphine | 5 |
| Butalbital | 100 | Naloxone | 1 |
| Carbamazepine | 200 | Norbuprenorphine | 0.5 |
| Carfentanil | 0.1 | Nordiazepam | 20 |
| Carisoprodol | 20 | Norfentanyl | 1 |
| Chlordiazepoxide | 5 | Norfluoxetine | 5 |
| Citalopram/escitalopram^2^ | 5 | Nortriptyline | 20 |
| Clomipramine | 20 | O-desmethylvenlafaxine | 10 |
| Clonazepam | 5 | Olanzapine | 5 |
| Cocaine | 20 | Oxazepam | 20 |
| Codeine | 5 | Oxycodone | 5 |
| Cyclobenzaprine | 1 | Oxymorphone | 5 |
| Desalkylflurazepam | 5 | Paroxetine | 5 |
| Desipramine | 20 | PCP | 5 |
| Dextromethorphan | 10 | Phenobarbital | 100 |
| Diazepam | 20 | Pregabalin | 100 |
| Diphenhydramine | 10 | Propanolol | 10 |
| Doxepin | 20 | Propoxyphene | 10 |
| EDDP | 20 | Pseudoephedrine | 5 |
| Fentanyl | 0.1 | Quetiapine | 50 |
| Flunitrazepam | 5 | Secobarbital | 100 |
| Fluoxetine | 5 | Sertraline | 5 |
| Flurazepam | 5 | Sufentanil | 0.1 |
| Gabapentin | 200 | Temazepam | 20 |
| Hydrocodone | 5 | THC-COOH | 5 |
| Hydromorphone | 5 | Topiramate | 200 |
| Imipramine | 20 | Tramadol | 10 |
| JWH-018 N-4-OH pentyl | 5 | Trazadone | 50 |
| JWH-018 pentanoic acid | 5 | Venlafaxine | 10 |
| JWH-073 N-3-OH butyl | 5 | XLR-11 | 5 |
| JWH-250 N-4-OH-pentyl | 5 | Zolpidem | 5 |
| Ketamine | 10 |  |  |

*This assay cannot distinguish amobarbital from pentobarbital
